# Anti-PD-1/PD-L1 Therapy for Non-Small-Cell Lung Cancer: Toward Personalized Medicine and Combination Strategies

**DOI:** 10.1155/2018/6984948

**Published:** 2018-08-08

**Authors:** Hongshu Sui, Ningxia Ma, Ying Wang, Hui Li, Xiaoming Liu, Yanping Su, Jiali Yang

**Affiliations:** ^1^Department of Histology and Embryology, Taishan Medical University, Taian, Shandong 271000, China; ^2^Ningxia Human Stem Cell Institute, General Hospital of Ningxia Medical University, Yinchuan, Ningxia 750004, China

## Abstract

Lung cancer remains a leading cause of cancer-related mortality worldwide with the poor prognosis. Encouragingly, immune checkpoint blockade targeting programmed death-1 (PD-1) and programmed death-ligand 1 (PD-L1) has dramatically changed the landscape for treatments in patients with non-small-cell lung cancer (NSCLC). However, only a small proportion of NSCLC patients responded to monotherapy of anti-PD-1/PDL1 agents; together, the development of resistance to anti-PD-1/PD-L1 therapy that leads to failure of anti-PD-1/PD-L1 therapy has significantly limited a broad applicability of the findings in clinical practices. Nowadays, several companion diagnostic assays for PDL1 expression have been introduced for identifying patients who may benefit the immunotherapy. In addition, results from clinical trials explored combinatory therapeutic strategies with conventional and/or targeted therapy reported a higher efficacy with an acceptable safety profile in NSCLC treatments, as compared to the monotherapy of these agents alone. In this review article, we summarized several anti-PD-1/PD-L1 agents licensed for NSCLC treatment, with a focus on predictive biomarkers and companion diagnostic assays for identification of NSCLC patients for immunotherapy anti-PD-1/PDL1 antibodies. Of a great interest, potentials of the combinatory therapy of anti-PD-1/PDL1 therapy with a conventional or targeted therapy, or other immunotherapy such as CAR-T cell therapy were emphasized in the article.

## 1. Introduction

Lung cancer is still main leading cause of cancer-related mortality worldwide with the poor prognosis. The non-small-cell lung (NSCLC) accounts for ~85% of all patients with lung cancer, and 15%–30% of NSCLC are lung squamous cell carcinoma (SQC) [1]. Over the past few decades, the conventional therapeutics (such as surgical resection, chemotherapy, and/or radiotherapy) has been used for treating advanced NSCLC patient. To date, the platinum-based chemotherapy still serves as the first-line therapeutic agent for lung cancer, with a median survival rate of approximately 9–12 months [2]. The therapeutic efficacy has been significantly improved with the introduction of targeted therapies, such as epidermal growth factor receptor tyrosine kinase inhibitors (TKIs) (gefitinib, erlotinib, afatinib, and osimertinib) and anaplastic lymphoma kinase (ALK) inhibitors alectinib, crizotinib, and ceritinib, as evaluated by studies including ALEX study, J-ALEX study [3, 4], ASCEND study [5, 6] PROFILE study [7], and ALUR study [8]. However, these targeted therapies only show excellent initial clinical responses to advance the lifetime of NSCLC patients; the development of resistance limits the therapeutic efficacy of these agents [9, 10]. Therefore, novel treatment strategies or agents are unmet need to improve the survival rate in NSCLC patients. Encouragingly, immune checkpoint blockade therapy is one of the most successful and exciting clinical benefits in advanced NSCLC [11].

Immune checkpoint inhibitor (ICI) is designed to target an inhibitory immune checkpoint molecule, such as programmed death-ligand 1 (PD-L1) and its receptor, programmed death-1 (PD-1), or cytotoxic T-lymphocyte-associated antigen 4 (CTLA-4) (Table 1) [12]. Agents targeting the PD-1/PD-L1 signaling have shown promising response in NSCLC treatment. Two antibodies (nivolumab and pembrolizumab) to PD-1 and two antibodies (atezolizumab and durvalumab) to PD-L1 have been approved by the US Food and Drug Administration (FDA) and/or European Medicines Agency (EMA) for treatment of NSCLC (Table 1) [13–16]. Unfortunately, only approximately 20% patients have positively response to ICIs as monotherapy for NSCLC. Therefore, it is of importance to identify patients who may benefit for immune checkpoint blockade therapy. Currently, four immunohistochemistry (IHC) assays (22C3, 28-8, E1L3N, and SP124) have been registered by FDA as companion and complementary diagnostic assays for detecting the expression of PD-L1 in practice (Table 2). The introduction of these assays has significantly increased the benefit of anti-PD-1/PD-L1 treatments [17], albeit many challenges in anti-PD-1/PD-L1 therapy remain to be overcome. In present review article, the characteristics of anti-PD-1/PD-L1 antibodies, biomarkers, and companion diagnostic assays for patient identification and the significance of the correlation between PD-1/PD-L1 signaling and other driver oncogenes (*EGFR*, *ALK*, *KRAS*, *MET*, *ROS1*) in a combinatory therapy including immune checkpoint blockades and targeted agents were also highlighted.

## 2. Companion and Complementary Diagnostic Assays for Precision Medicine of PD-1/PD-L1 Inhibitors

**Figure 1:**
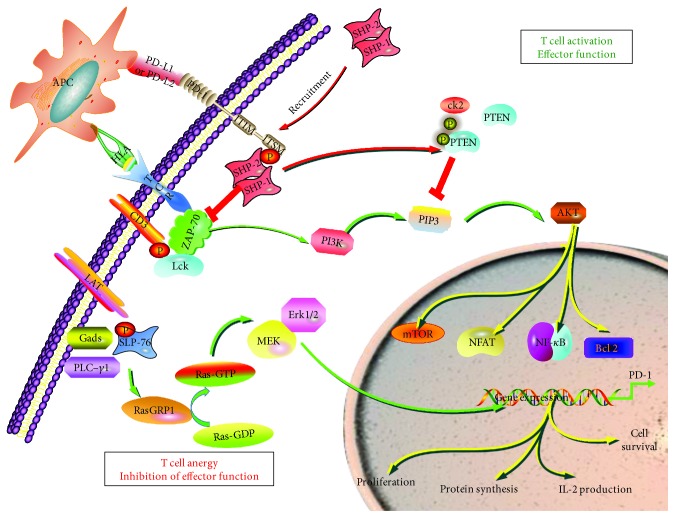
Effect of PD-1/PD-L1 signaling on major signaling pathways and reprograming in T cells. Upon the stimulation of antigen, the MHC on the surface of APC could present antigens to the TCR and promote TCR/CD3 chains to phosphorylate, resulting in an activation and recruitment of Lck and Zap-70, which in turn lead to the phosphorylation of tyrosine motifs (ITAM) and initiation of the downstream signaling cascade of TCR. However, in the pathological state, the PD-1 bind to its ligand PD-L1 or PD-L2; the tyrosine phosphatase SHP-2 or SHP-1 can be recruited and bind to the ITSM sequence in the PD-1 cytoplasmic tail. An activation of PD-L/PD-L1 signaling PD-1 mediates the inhibition of the PI3K/Akt and Ras/MEK/Erk signaling pathway, resulting in the inhibition of T cell proliferation, protein synthesis, survival, and IL-2 production. APC: antigen-presenting cell; HLA: human leukocyte antigen; TCR: T cell receptor.

In the tumor environment, a binding of PD-1 and PD-L1 suppresses the activated T cell proliferation, cytokine release, and cytolytic activity of PD-1-positive T cells and promotes tumor cell escaping from host immune attack [18]. Therefore, targeting PD-1/PD-L1 signaling can enhance the capacity of activated T cells to recognize and kill tumor cells and subsequently, restore the function of host immune surveillance, by the activation of PI3K/Akt and Ras/MEK/Erk signaling pathways (Figure 1) [19, 20]. Given its important role in immunosuppression, the immune checkpoint signaling has been utilized as novel targets for developing antitumor agents.

Nowadays, several anti-PD-1/PD-L1 antibodies have been licensed for treatments of many types of solid tumors in clinical settings, including the NSCLC (for details see Table 1) and many are in developed. Among four of them, nivolumab (Opdivo) [21], pembrolizumab (Keytruda) [16], atezolizumab (Tecentriq, MPDL3280A, RG7446), and durvalumab (Infinzi) [22] have been approved for patients with NSCLC.

Despite the drug blockading the PD-1/PD-L1 pathways has been shown exciting therapeutic benefits for patients with advanced NSCLC, less than 20% NSCLC patients could benefit for these novel agents [23–25], suggesting an unmet need for identification of patients in this type of immunotherapy. Indeed, clinical trials have showed that examining of PD-L1 by IHC assays may help in guiding NSCLC patients to choose agents.

In this respect, at least six antibodies are currently used to assay PD-L1 expression. These agents all have a biomarker assay linked to their use. However, only the 22C3 pharmDx assay has status as a companion diagnostic for use of pembrolizumab. The PDL1 IHC 22C3 pharmDx assay and Ventana PD-L1 (SP142) assay have status as complementary diagnostics for nivolumab and atezolizumab, respectively [26, 27].

In 2015, two IHC assays of PD-L1 together with their corresponding agents for NSCLC patients have been authorized for by FDA and/or EMA: one is PD-L1 IHC 22C3 pharmDx assay and the other is 28-8 assay. The Dako 22C3 IHC assay is a companion diagnostic assay for pembrolizumab (Keytruda) (https://www.accessdata.fda.gov/cdrh_docs/pdf15/P150013S006A.pdf), and the 28-8 assay is a complementary diagnostic for nivolumab (Opdivo) (https://www.accessdata.fda.gov/cdrh_docs/pdf15/p150027c.pdf). Similarly, the SP142 PD-L1 IHC assay is developed as the complementary diagnostic with atezolizumab (Tecentriq) (https://www.accessdata.fda.gov/cdrh_docs/pdf16/p160002c.pdf), and Ventana SP263 is developed with durvalumab (Imfinzi) (https://www.accessdata.fda.gov/cdrh_docs/pdf16/p160046c.pdf). Both of SP142 and Ventana SP263 assays are developed by Ventana Medical System for Roche (Roche, Tucson, AZ, USA). The 78-10 antibody, however, was developed to company with avelumab (Bavencio) [28]. These three assay platforms are developed by Dako Company (Current Agilent, Santa Clara, CA, USA). In addition, a diagnostic IHC assay using E1L3N antibody has also been developed for both the Dako and Ventana platforms [28, 29].

Clinically, PD-L1 IHC 22C3 pharmDx is a qualitative IHC assay for NSCLC tissue *in vitro* diagnostic and helps to identify NSCLC patients for curing with pembrolizumab, which applies monoclonal mouse anti-PD-L1. The antibody produced by clone 22C3 was able to recognize and bind to the PD-L1 protein in formalin-fixed, paraffin-embedded (FFPE) NSCLC tissue. The sections are stained with monoclonal mouse anti-PD-L1 or the negative control reagent (NCR) by using an EnVision FLEX visualization system on the Autostainer Link 48 system. The level of PD-L1 protein expression was evaluated by the standard of tumor proportion score (TPS). The PD-L1-positive cells for this assay ranged from as low as 1% to as high as 50%. The specimen should be considered PD-L1-positive if the TPS of viable tumor cells with membrane staining at any intensity was ≥1% (Table 2) [30]. A previously treated patient with ≥1% PD-L1-positive cells in tumor might benefit from pembrolizumab as a secondary line therapy, and a previously untreated patient with ≥50% PD-L1-positive cells in tumor might benefit from pembrolizumab as the first-line therapy [26].

PD-L1 IHC 28-8 pharmDx is also an IHC assay for the diagnosis of IHC in clinical trials with nivolumab, by detecting PD-L1 protein in FFPE section of nonsquamous NSCLC and melanoma tissues. The sections are stained with monoclonal rabbit anti-PDL1 or NCR by using an EnVision FLEX visualization system on the Autostainer Link 48 system to visualize the result. A ≥1, 5, and 10% of PD-L1-positive cells in NSCLC tumors detected by this assay were recommended in various clinical trials [26].

Ventana PD-L1 (SP142) assay is a diagnostic assay complementary to treatment using atezolizumab. It is a qualitative immunohistochemical assay using rabbit monoclonal primary antibody to recognize PD-L1 in paraffin-embedded tissue sections of NSCLC and urothelial carcinoma. In NSCLC, a patient who was detected with a PD-L1 expression ≥ 50% in tumor cells or ≥10% in immune cells would be eligible for treatment with atezolizumab [26, 29].

Similarly, Ventana PD-L1 (SP263) assay is another PD-L1 protein detecting system developed for NSCLC and other tumor types by the Ventana BenchMark ULTRA [31]. It has been tested as a companion assay for employment of nivolumab, pembrolizumab, and durvalumab. Of note, the SP263 assay could expand the therapeutic range for NSCLC patients. In this regard, identified NSCLC patients can use pembrolizumab and identified nonsquamous NSCLC patients can select nivolumab. For pembrolizumab, patients who were detected a ≥50% PD-L1-expressing tumor cells in NSCLC tissues could be recommended for a first-line therapy, while those who had a ≥1% PD-L1-positive tumor cells are for a second-line treatment. Noteworthy, a ≥1, 5, and 10% of PD-L1-positive cells in NSCLC tumors detected by SP263 were reported in different clinical trials [29, 31]. The introduction of PD-L1-based companion and complementary diagnostic assays has greatly increased the efficacy and safety of anti-PD-1/PD-L1 therapy in selected patients, suggesting that the PD-L1 is one of the predictive biomarkers for PD-1/PD-L1 inhibitors in NSCLC.

In order to determine technical equivalences of the above four IHC assays and alterations of antibodies on other staining platforms, comparisons of these PD-L1 IHC assays in NSCLC have been performed by several groups [32, 33]. The study by Fujimoto et al. suggested an equivalent predictive performance of 28-8, 22C3, and SP263 PD-L1 IHC assays, while the SP142 assay exhibited a lower predictive performance in NSCLC [32]. However, results from the study of Hendry et al. reported that only the 28-8 and 22C3 IHC PD-L1 assays showed a concordant performance and could be used interchangeably in clinical settings [33].

## 3. PD-L1 as a Predictive Marker of Response to PD-1/PD-L1 Inhibition in NSCLC Treatment

A compelling body of evidence has shown that anti-PD-1/PD-L1 therapy is a promising treatment strategy with unprecedented survival benefits for selected NSCLC patients. Nevertheless, a substantial proportion of patients who received PD-1/PD-L1 inhibitors have little or no benefit. Therefore, it is a need to identify biomarkers for establishment of valid predictors of treatment responses [34]. In this regard, the expression of immunosuppressive molecules including PD-L1, PD-1, and indoleamine 2,3-dioxygenase (IDO), as well as mutational landscape and mutational load [35] and mismatch repair deficiency (MMRD) [36], has been examined as potential predictor of response to anti-PD-1/PD-L1 treatments. Among them, the expression of PD-L1 in tumors has been employed as the criteria in some of the studies of anti-PD-1/PD-L1 therapy for NSCLC.

Indeed, several clinical trials have reported remarkable results of therapeutic outcome of immune checkpoint blockades in NSCLC patients guided by PD-L1 IHC assays [11, 12]. Currently, aforementioned four commercial IHC-based bioassays are employed as diagnostic assays for detecting PD-L1 expression of tumors to guide the use of PD-1/PDL1 inhibitors alone or in combination with other therapeutic agents with different cutoff values in distinct types of lung cancer (for details see Table 2).

Durvalumab (Imfinzi, MEDI4736) is a human IgG1 anti-PD-L1 mAb, which showed a potential to improve progression-free survival in patients with locally advanced, unresectable stage III NSCLC, which met its primary endpoint in the PACIFIC trial for assessing its effects in patients with locally advanced NSCLC after standard chemoradiotherapy [37, 38]. In addition, its potential may be strengthened if the ongoing phase III randomized studies of first line (NCT02453282, MYSTIC trial) and second or subsequent (NCT02352948, ARCTIC trial) lines of therapy demonstrate superiority over the current standard of care [39]. Thus, it was approved for treatment of patients with locally advanced, unresectable stage III NSCLC who do not relapse after platinum-based chemoradiation on Feb. 16, 2018. Its optimal clinical role in managing lung cancer remains unclear, and more robust predictive biomarkers are needed, although the Roche Ventana SP263 has been used as a diagnostic assay for treatment with durvalumab [37, 38]. In addition, the Roche Ventana SP263 assay is used in an ongoing phase III study of durvalumab monotherapy in patients with PD-L1 positivity (≥25% tumor cells stained using Roche Ventana SP263) and the durvalumab combined with or without tremelimumab (ARCTIC trial) or either agent versus standard of care (SoC) (such as erlotinib, gemcitabine, and vinorelbine) in patients with PD-L1 tumors [39]. However, AstraZeneca and MedImmune recently reported that the ARCTIC trial did not meet the primary endpoint of improving PFS compared to SoC in NSCLC patients who had a 25% or more of tumor cells expressing PD-L1 in tumor as determined by the Ventana PD-L1 SP263 assay. The trial is ongoing to assess two additional primary endpoints of overall survival (OS) for durvalumab monotherapy and OS for the durvalumab and tremelimumab combination.

Atezolizumab was approved for second-line treatment of patients with advanced NSCLC based on PD-L1 expressing in tumor tissues. In the phase II trial of atezolizumab as first-line or subsequent therapy in advanced NSCLC patients selected with PD-L1 (BIRCH) (NCT02031458), 3914 NSCLC patients were enrolled in accordance with PDL1 expression in tumor-infiltrating immune cells (IC) or tumor cells (TC) and received atezolizumab at a dose of 1200 mg every 3 weeks. The TC PD-L1 positive was defined as TC3 ≥ 50% or TC2 ≥ 5% but <50%, and the IC PD-L1 positive was defined as IC3 ≥ 10% or IC 2 ≥ 5% but <10%, as determined by the PD-L1 expression using the SP142 immunohistochemistry assay. This phase II study was divided into three groups: cohort 1 (without chemotherapy advanced NSCLC, 1L), cohort 2 (first-line platinum-containing chemotherapy advanced late NSCLC, 2L), and cohort 3 (at least 2-line chemotherapy advanced late NSCLC, ≥3L). The one-year ORR was 22%, 19%, and 18% for cohorts 1, 2, and 3, respectively; and the ORR in the TC3 or IC3 subgroup for respective cohorts 1, 2, and 3 was 31%, 26%, and 27%. The OS in cohorts 1, 2, and 3 was 23.5 (26.9 months for TC3 or IC3 patients), 15.5, and 13.2 months, respectively. This study showed that the atezolizumab monotherapy was safe and tolerable for PD-L1-selected advanced NSCLC patients [40].

Of note, these PD-L1 IHC assays showed similarities and differences in clinical trials. In order to better unravel the reliability of these IHC assays in patient selection for anti-PD-1/PD-L1 therapy, the Blueprint PD-L1 IHC Assay Comparison Project was founded (AACR, 2015) [17, 31]. In this project, Rimm et al. and Scheel and Schäfer compared five PD-L1 IHC assays (22C3, 28-8, SP142, SP263, and 73-10) [17, 31]. In the clinical phase I trial, they observed that 22C3, 28-8, and SP263 assays were closely aligned on tumor cell staining, whereas the SP142 assay showed fewer stained tumor cells in 19 of 38 samples (50.0%). Nonetheless, NSCLC tumors were classified using respective criteria of the above 4 assays; 5 of 38 (13%) were determined below the selected cutoffs of all assays. By comparing assays and cutoffs, this data demonstrated that the analytical performance of PD-L1 expression was comparable among 22C3, 28-8, and SP263 assays. A change of assays and cutoffs would lead to “false classification” of PD-L1 states in some patients. Therefore, more data is needed to inform the use of alternative staining assays on PD-L1 cutoff values for different specific therapies [31].

Interestingly, SP142 antibody showed an outlier that has a significantly less mean score of the PD-L1-positive rate in both tumor and immune cells (tumor cells: 1.99; immune cell: 1.62) relative to other three antibodies (tumor cells: 22c3, 2.96; 28-8, 3.26; E1L3N, 3.20; and immune cells: 22c3, 2.15; 28-8, 2.28; E1L3N, 2.28) in this study. In addition, the intraclass correlation coefficients (ICCs) of the pathologist score and concordance suggested that pathologists have significant concordance for scores of PD-L1 expression in tumor cells (ranged from ICCs of 0.832 (95% CI, 0.820–0.844) to 0.882 (95% CI, 0.873–0.891)), but there were poor concordance of ICC immune cells stained with any antibody ranged from 0.172 (95% CI, 0.156–0.189) to 0.229 (95% CI, 0.211–0.248) [17, 31]. In the same project, Rimm et al. compared the PD-L1 expression in 90 of stages I–III NSCLC samples using four IHC assays (22C3, 28-8, SP142, and E1L3N) [17]; the result was similar to the above study [31]. Together, these results indicated that 22C3, 28-8, SP263, and E1L3N were better assays for PD-1 expression in clinical settings. Recently, several lines of studies in PD-L1 IHC assays for NSCLC using 22C3 and 28-8 further supported the results of the above two studies [31] [41].

In order to unravel the relationship between PD-L1 expression and NSCLC patient subsets, Igawa et al. accessed the expression of PD-L1 in 229 consecutively resected NSCLC specimens using SP263 immunohistochemical assay and found that the PD-L1 expression was significantly associated with male patients or current smokers [41]. Moreover, a higher PD-L1 expression was determined in squamous NSCLC samples as compared with nonsquamous NSCLC samples (53 and 71%, respectively; *P* = 0.026) [41]. In addition, the high expression of PD-L1 was associated with a low 5-year survival rate in squamous NSCLC [41]. Aforementioned studies thus suggest that an identification of subtypes of NSCLC is the first step in PD-1/PD-L1 therapy for precise treatment.

In addition, PD-L1 may be also as a biomarker in stage III/IV lung squamous cell carcinoma (SQC). A preliminary study of Chinese patients with stage III/IV lung SQC showed that PD-L1 expression was detected in 61.7% (79/128) of patients. Interestingly, more abundant PD-L1 were observed in samples of smokers over nonsmokers (66.0% versus 44%, *P* = 0.042, resp.). However, a combination of PD-L1 expression and clinicopathologic parameters showed no significant difference in the objective response rate (ORR) for platinum-based chemotherapy between PD-L1-negative and positive patients (43.3% versus 36.2%, *P* = 0.434; 80.0% versus 78.7% *P* = 0.840, resp.), the overall survival (OS) in PD-L1-negative patients was significantly lower than PD-L1-positive patients (41.5 versus 19.3 months, *P* = 0.001) [42]. This study indicated that the PD-L1 was a potential biomarker for anti-PD-1/PD-L1 therapy in smoking-related lung SQC.

Despite an increased number of clinical trials has demonstrated that the PD-L1 is a valuable biomarker for guiding anti-PD-1 and anti-PD-L1 treatments in various cancers [42], the correlation between the PD-L1 expressions determined by an IHC assay could not been reproduced across various studies and tumor subtypes. For instance, the PD-L1 expression was clearly correlated with the clinical response to treatment of nivolumab and pembrolizumab for nonsquamous NSCLC [23, 24], but not correlated with the response in squamous cell NSCLC [23]. Moreover, the PD-L1 expression in immune effector cells of tumor microenvironment is also a potential predictor [43, 44]. For example, the reservoir of PD-L1-negative TILs provided an immune-privileged microenvironment with a positive impact on survival of patients with resected disease and response to nivolumab in advanced NSCLC patients with intrinsic variability of PDL1 expression in tumors [45]. Currently, there is no standard of PD-L1 IHC assay that has been recommended in a guideline of cancer therapy using immune checkpoint blockades. Therefore, there is a need to harmonize the available PD-L1 assays in clinical practice. Nonetheless, the introduction of PD-L1 IHC assays in anti-PD-1/PD-L1 therapy has dramatically increased the therapeutic benefits in NSCLC patients, suggesting an importance of PD-1/PD-L1 as a predictive biomarker for precision medicine in NSCLC using anti-PD-1/PD-L1 therapy (Table 2).

In addition to the PD-L1 as a testing biomarker for anti-PD-1/PD-L1 therapy in NSCLC, several other biomarkers were identified for patient selection in checkpoint blockade therapy. These included tumor mutational load, the “inflamed phenotype” such as TILs and immunoscore, TCR clonality, gene signatures, plasma biomarkers such as cytokines [46], and most recently, the tumor mutation burden (TMB) [47]. For instance, NSCLC patients with high nonsynonymous mutation burden showed more durable clinical benefit to treatment with pembrolizumab with a higher PFS and ORR relative to those with less frequent nonsynonymous mutations [35]. Another example is the serum concentration of IL-8 that is also a surrogate biomarker for predicting response in melanoma and NSCLC patients treated with pivolumab or pembrolizumab alone or nivolumab plus ipilimumab [48]. Of great interest, high TMB is a poor prognostic factor in NSCLC [47], a most recently whole-exome sequencing (WES) study of NSCLC patients treated with PD-1 plus CTLA-4 blockade; however, the study demonstrated that TMB was independent of PD-L1 expression and was the strongest feature associated with improved objective response, durable benefit, and PFS in PD-1 plus CTLA-4 combination of immunotherapy in NSCLC [49]. These findings suggest that high TMB is a predictor of the efficacy of immune, which may be an independently predictive marker for benefits of combination immunotherapy in NSCLC with PD-L1 and CTLA-4 blockade in future clinical trials [47, 49].

## 4. PD-1/PD-L1 Signaling Pathway as a Therapeutic Target for NSCLC

Targeting the PD-1/PD-L1 signaling pathway has recently become a promising therapeutic strategy in cancer therapy. Such a strategy is through a mechanism by preventing the escape of tumor cells from the immune surveillance and restoring the function of the host's immune system to attack the tumor cells (Tables 1 and 3) [11].

The encouraging clinical safety and effects of anti-PD-1/PD-L1 antibodies in both clinical trials and settings have led a fast development of inhibitors of PD-1 and PD-L1/2 for treatments of solid tumors, including the NSCLC. In this regard, nivolumab was firstly assessed for its safety and clinical activity in 39 patients with intractable solid tumors (including advanced metastatic NSCLC, melanoma, colorectal cancer (CRC), castrate-resistant prostate cancer, or renal cell carcinoma (RCC)) [50]. Subsequently, a dose-increasing trial of multiple doses of nivolumab was conducted in 296 patients with advanced malignancies, including 122 patients with NSCLC (with or without *EGFR* or *KRAS* mutation, squamous or nonsquamous type, PD-1 expression positive or negative) [51]. This objective response rates (ORRs) were 28% (26 of 94 melanoma patients), 27% (9 of 33 RCC patients), and 18% (14 of 76 NSCLC patients) (NCT00730639) [51]. Responses were observed for at least 1 year in the great mass of patients. Furthermore, preliminary data indicated that the objective response was correlated with PD-L1 expression of cancer tissue as determined by immunohistochemical analysis [51]. Meanwhile, the phase I study using another anti-PD-L1 monoclonal antibody (BMS936559) was demonstrated to give rise to durable tumor regression with the ORR ranging from 6% to 17% in advanced cancers, including 75 with non-small-cell lung cancer (NCT00729664) [52]. On the basis of these phase I results, several randomized, open-label, and international phase 3 trials were initiated [23, 53, 54]. One study was designed to compare nivolumab with docetaxel in advanced nonsquamous NSCLC [53]. In this study, the patient group treated with nivolumab resulted in advancing median overall survival (OS) compared to docetaxel (OS 12.2 versus 9.4 months); the OS at one year was 51% (95% CI, 45 to 56) in patients treated with nivolumab versus 39% (95% CI, 33 to 45) in the docetaxel group (NCT01673867) [53]. At the same time, the phase III study by Brahmer et al. followed the same design and showed an increased OS in the nivolumab group compared to the docetaxel group (9.2 versus 6.0 months, resp.) (NCT01642004). Unexpectedly, the expression of PD-L1 in tumor determined at three different expression cutoff values (1, 5, and 10%) showed neither prognostic nor predictive treatment benefit in this study [23]. However, a randomized phase II study in investigation of the efficacy and safety of atezolizumab comparing with docetaxel in previous platinum-treated NSCLC patients (who were PD-L1-positive as determined by SP142 antibody IHC assay) showed that atezolizumab resulted in an enhanced PFS (9.7 versus 3.9 months) and OS compared to docetaxel (12.2 versus 9.4 months) for patients with high levels of PD-L1 expression (NCT01903993) [54].

The promising results of clinical trials led FDA to approve nivolumab for treatment of intractable metastatic squamous NSCLC in 2015. Almost at the same time, another anti-PD-1 antibody, pembrolizumab, was licensed for treatment of PD-L1-positive advanced NSCLC by FDA/EMA, based on a mass of clinical trials exhibited that pembrolizumab was safe and effective for metastatic NSCLC (mNSCLC) [14, 15]. Moreover, the cutoff > 50% PD-1-positive patients exhibited a better benefit from pembrolizumab therapy [14]. It therefore was approved for first-line treatment of patients with mNSCLC whose tumors have high PD-L1 expression (tumor proportion score (TPS) ≥ 50%) and wild type of *EGFR* or *ALK* genes [55]. This approval expanded the indication of pembrolizumab as a second-line treatment of lung cancer. Indeed, meta-analysis in randomized clinical trials of anti-PD-1/PD-L1 therapy (atezolizumab, pembrolizumab, and nivolumab) demonstrated the efficacy and safety and could dramatically improve the PFS and OS compared with docetaxel for patients with previously treated NSCLC [56, 57] and enhance the PFS and OS compared to EGFR-TKI alone for NSCLC patients with *EGFR* wild type [56]. Of note, as far as PFS was a concern, an anti-PD-1/PD-L1 therapy was second only to EGFR-TKI therapy for patient with *EGFR* mutation [56]. These clinical trials clearly demonstrated that inhibition of the PD-1/PD-L1 pathway could clinically improve the ORR, OS, and PFS compared with single-arm chemotherapy alone in nonsquamous and squamous NSCLCs. In addition, these data also showed that PD-1 or PD-L1 could serve as a biomarker to improve the benefit of anti-PD-1/PD-L1 therapy for NSCLC patients.

## 5. Anti-PD-1/PD-L1-Based Combinatory Therapies for NSCLC Treatment

Owing to the lack of a definite biomarker for the selection of patients who likely benefit from checkpoint inhibitor-based monotherapy, anti-PD-1/PD-L1-based combinatory therapies were designed. In addition, the primary or acquired resistance to immune checkpoint inhibitors and/or conventional therapies is inevitable in most cases of NSCLC. Therefore, combinatory therapies designed to reenergize the immune system with complementary/synergetic mechanisms were introduced as an alternative strategy for NSCLC treatment in clinical settings. These studies are on the basis of anti-PD-1/PD-L1 agents with other immunotherapies (such as CTLA-4 blockade), chemotherapy, radiotherapy, and targeted therapy [44, 49, 58–62].

A compelling body of studies has demonstrated that the PD-L1 expression was associated with *EGFR* mutation, *ALK* rearrangements, or *KRAS* mutation in NSCLC [21, 63–67]. These studies evidenced that oncogene drivers could enhance immune escape of tumor cells by upregulating PD-L1 expression in NSCLC. For example, the PD-L1 expression was linked to *KRAS* mutations and was significantly associated with *EGFR* mutations [68]. Moreover, an activation of the oncogenic *EGFR* signaling pathway could enhance PD-L1 expression and suppress antitumor immunity [69]. These studies suggest that a combination of PD-1/PD-L1 blockades with EGFR-TKIs may enhance the outcome of NSCLC treatment. In this respect, a combination of nivolumab with erlotinib showed an increased OS, ORR, and PFS in NSCLC patients with acquired resistance to erlotinib [70]. The combinatory therapy of durvalumab and gefitinib (NCT02088112) [71] and durvalumab (NCT0214346) [72] also showed an increased ORR in NSCLC patients with *EGFR* mutation. However, the combination of durvalumab and osimertinib (AZD9291) has been held due to the unacceptably high rate of pneumonitis.

In addition, PD-L1-positive patients exhibited more sensitive to gefitinib or erlotinib than PD-L1-negative patients in terms of TTP and OS [68]. Interestingly, an acquired resistance to gefitinib could enhance the PD-L1 expression and *MET* positivity in *EGFR*-mutant NSCLC [73]. Similarly, NSCLC patients harboring mutant *KRAS* treated with immune checkpoint inhibitors (ICIs) had prolonged OS relative to those containing wild-type KRAS [65]. These results implied that high mutational rates of *EGFR* or *KRAS* mutations enhanced immunogenicity and could serve as potential biomarkers for anti-PD-1/PD-L1 therapy [65, 69]. These findings were supported by recent whole-exome sequencing (WES) studies in NSCLC, in which patients with higher TMB benefited more from a combination immunotherapy in NSCLC [47, 49].

Indeed, NSCLC patients with acquired resistance of EGFR-TKIs and *EGFR* mutation but not T790M were reported to benefit more from nivolumab than those with T790M mutation [74]. In this study, 25 patients with *EGFR* mutation-positive NSCLC (cohort A) and 60 patients with acquired EGFR-TKI resistance who were included in whole-exome sequencing (cohort B) were treated with nivolumab. The results displayed that the median PFS of patients with the T790M-negative PD-L1 expression level of ≥1% and those who had a T790M-positive PD-L1 expression level of <1% were 2.1 and 1.3 months, respectively. Generally, the PD-L1 expression of ≥10% or ≥50% was associated with PFS in T790M-negative patients. These results warrant further investigation for prospective outcomes of clinical trials [74]. Further studies on the relationship between driver oncogene mutations (*EGFR*, *ALK*, *KRAS*, *MET*, and *ROS1*) and immune-related biomarkers (PD-1, PD-L1, CTLA-4, and CD8) in NSCLC demonstrated that the PD-L1 expression of 26% of SCC and 76% of adenocarcinoma samples was overlapped with driver oncogenes [75], but a TPS of PD-L1 ≥ 50% was rarely overlapped with driver oncogenes [76]. Interestingly, frequent PD-L1/CD8-double positive TILs were observed in TPS [75], and high density of CD8-positive TIL and nonsynonymous mutation burden were found in nivolumab responders [74]. To date, several clinical trials of a combination of anti-PD-1/PD-L1 agents (atezolizumab, pembrolizumab, and nivolumab) with EGFR-TKIs (gefitinib and erlotinib) for NSCLC treatment are undergoing (Table 3), and these are identified as a major breakthrough in advanced or previously treated advanced NSCLC. It is worthy to note that the relationship of PD-L1 expression and *EGFR* oncogenic mutations is controversial between different studies. Both low and high *EGFR* mutations with PD-L1 expression were reported [77]. In addition, treatments of chemoradiation and TKIs might also alter the PD-L1 expression.

Chemotherapy, particularly the platinum-based doublet chemotherapy (PT-DC) is the first-line treatment for patients with advanced NSCLC. Interestingly, an increased number of evidences unraveled that chemotherapy has an impact on immune microenvironment of tumors, which in turn enhances its antitumor, through mechanisms including reduction of T-regulatory cell activity, selective depletion of myeloid-derived suppressor cells (MDSCs), induction of PD-L2 expression, and the maturation of APCs [78, 79]. Therefore, a combination of anti-PD-1/PD-L1 therapy with standard chemotherapy may result in a synergistic antitumor activity for NSCLC treatment. Indeed, a growing body of clinical studies has shown encouraging outcomes for combination therapies of each of three approved anti-PD-1/PD-L1 agents, pembrolizumab, nivolumab, and atezolizumab with chemotherapy in NSCLC. The result of phase II randomized trial of pembrolizumab plus chemotherapy (carboplatin and pemetrexed) showed a 55% of ORR, which was significantly increased in comparison with the 29% ORR in treatment with first-line chemotherapy alone for advanced nonsquamous NSCLC [80]. Similarly, results from the CheckMate 012 trial also showed a range of 33%–47% of ORR in first-line treatment of combinatory therapy of nivolumab and chemotherapy for advanced NSCLC [81]. Similarly, the combination of atezolizumab with platinum-based doublet chemotherapy resulted in a promising activity (ORRs: 60%–75%) with no unexpected toxicities as first-line therapy for locally advanced or mNSCLC (NCT01633970) [82]. Of note, the sample sizes of the above studies were small and lack of data of the improvement of PFS and OS. In addition, the adverse effect of the combination therapy of immune checkpoint inhibitors with chemotherapy was a safety concern. Therefore, a precise dosing regimen and ingenious strategy design are of importance to exert maximum antitumor activity with tolerable toxicity. Nevertheless, the robust antitumor activity and reliable safety profile of immune checkpoint inhibitor-combined chemotherapy motivated more phase III studies to investigate the efficacy and safety of a regimen including an immune checkpoint blockade for the first-line treatment of advanced NSCLC, and more randomized, double-blind, large cohort, phase III studies are planned or undergoing. Encouragingly, the study of Keynote-189 (NCT02578680) phase III trial recently reported that the combination of pembrolizumab and standard chemotherapy of pemetrexed and a platinum-based drug yielded a significantly longer OS and PFS over chemotherapy alone in metastatic NSCLC [83]; in addition, the IMpower150 (NCT02366143) phase III trial also showed a remarkable improvement of the OS and PFS of NSCLC patients treated with a combination of atezolizumab and bevacizumab plus carboplatin and paclitaxel significantly, compared to bevacizumab plus carboplatin and paclitaxel alone [84].

Similar to chemotherapy, radiotherapy is another common nonsurgical treatment for NSCLC. In addition to its ability to directly kill tumor cells, radiation is also able to trigger local immune responses and subsequently render the tumor microenvironment to recruit effective T cells [85]. However, the radiation-triggered immune response is far to generate a systemic antitumor immunity. Intriguingly, immune checkpoint inhibitors exhibit a capacity to enhance the radiotherapy-triggered local immune response to systemic antitumor effects, that is, abscopal effects [79, 86, 87]. Indeed, substantial data have shown that the addition of immune checkpoint inhibitor increased the effect of either radiotherapy or immunotherapy alone [88, 89]. In this respect, the ablative and highly targeted doses of stereotactic ablative radiotherapy (SABR) in combination with anti-PD-1/PD-L1 therapy have spurred an increased interest, since SABR can induce more robust immune response and reduce surrounding normal tissue toxicity than conventionally fractionated radiotherapy [90]. Of importance, an administration of PD-1/PD-L1 inhibitor before or during the radiotherapy is a more reasonable approach to bring a long-term antitumor effect than the immune checkpoint inhibitors which are given after the radiotherapy, as the concurrent chemoradiation therapy (CCRT) may induce the expression of PD-L1 [91, 92]. However, recent PACIFIC trial showed remarkable efficacy of durvalumab when it was administered following the completion of CCRT [44, 93], suggesting that further trials are required to optimize therapeutic regimens in combination of immune checkpoint blockades and CCRT. Indeed, several lines of clinical studies of anti-PD-1/PD-L1 therapy in combination with radiotherapy in the treatment of NSCLC are currently undergoing [94].

It has been demonstrated that angiogenic factors are immunosuppressive, implying that a combination of immune checkpoint blockade with antiangiogenic agents may exhibit synergistic antitumor activity in NSCLC treatments. Indeed, primary data from clinical trials in combination therapy with immune checkpoint blockade (nivolumab and pembrolizumab) and antiangiogenic agents (bevacizumab, ramucirumab, and nintedanib) show promising results for NSCLC [60]. For example, the phase I trial (NCT01454102) evaluated the efficacy and safety of combination maintenance treatment of nivolumab with bevacizumab in advanced NSCLC patients with response to first-line platinum-based chemotherapy. Preliminary results reported an acceptable toxicity profile of combination treatment with median PFS of 37.1 weeks in nonsquamous patients, but the nivolumab monotherapy showed the respective median PFS of 21.4 and 16 weeks for nonsquamous and squamous NSCLC patients [38]. Several other trials exploring a combination of immune checkpoint blockade and antiangiogenic agents are underway, including the combination of ramucirumab with pembrolizumab (NCT02443324) in patients with advanced solid tumors including NSCLC [95], pembrolizumab with nintedanib (NCT02856425) in advanced NSCLC [96], and bevacizumab plus chemotherapy with atezolizumab (NCT02366143) or pembrolizumab (NCT02039674) in the first-line setting for NSCLC treatment (Table 3) [60]. Remarkably, the phase III IMpower150 (NCT02366143) study has showed that the combination of atezolizumab and bevacizumab plus carboplatin and paclitaxel significantly improved the OS and PFS in NSCLC patients, compared to bevacizumab plus carboplatin and paclitaxel alone [84].

Indoleamine 2,3-dioxygenase (IDO) is a normal endogenous mechanism of acquired peripheral immune tolerance in vivo and is highly expressed in several cancer types and usually associated with poor prognosis. Trials evaluated anti-PD-1/PD-L1 or CTLA-4 antibodies (nivolumab, pembrolizumab, atezolizumab, and durvalumab) in combination with indoximod, BMS-986205, or epacadostat; inhibitors of IDO1 are underway (NCT02327078, NCT03085914, NCT02178722, NCT02862457, NCT02298153, NCT02318277, and NCT02658890) [97]. The phase I trial examined the combination of epacadostat with pembrolizumab in NSCLC patients exhibited a disease control rate of 58%, with a good safety profile, regardless of PD-L1 status [98, 99].

Apart from combinatory therapies with chemo-, radio-, or targeted therapy, the anti-PD-1/PD-L1 therapy combined with other immunotherapies also is an attractive approach for NSCLC treatment [100–102]. For example, both PD-1/PD-L1 and CTLA-4 checkpoint inhibitors are capable of enhancing antitumor T cell activity with different complementary mechanisms. It is therefore an anti-PD-1/PD-L1 therapy in combination with the anti-CTLA-4 agent may be a potential to improve the antitumor responses of each agent alone. However, clinical studies assessed the combination of durvalumab (anti-PD-L1) and tremelimumab (anti-CTLA-4) or pembrolizumab (anti-PD-L1) and ipilimumab (anti-CTLA-4) for treatment of NSCLC yielded no better ORR but increased AEs [38, 103, 104], despite encouraged response to the combination of nivolumab and ipilimumab was observed in NSCLC patients with high PD-L1 expression [37, 38]. The results from the undergoing phase III CheckMate 227 trial (NCT02477826) may offer the more informative data on the combinatory therapy of nivolumab plus ipilimumab in first-line treatment for advanced NSCLC [100].

**Figure 2:**
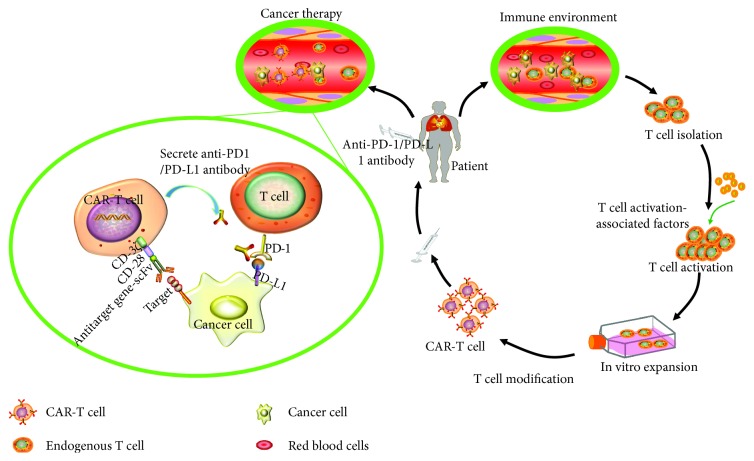
Illustrative image describing anti-PD-1/PD-L1 therapy combined with CAR-T cells. T cells are obtained and isolated from the patients. These T cells are transformed with chimeric antigen receptor (CAR) gene by lentiviruses. The CAR-T cells are expanded in vitro and finally infused back to patients. After the CAR-T cells transferred into patients, the CAR-T cells are able to recognize target gene-positive tumor cells. Meanwhile, CAR-T cells can secrete anti-PD-1/PD-L1 antibody and combine with foreign anti-PD-1/PD-L1 antibody to recognize and kill the tumor cells.

Interestingly, chimeric antigen receptor (CAR) T cell (CAR-T) therapy has demonstrated a promising clinical effect in broad of malignancies including chronic lymphoid leukaemia and lymphomas, despite it still faces a series of challenges in treatments of solid tumors. Encouragingly, recent studies unraveled that PD-1/PD-L1 inhibitors could increase the efficiency of T cell-based immunotherapy [105]. A previous study found that an antigen-specific stimulation of PD-L1(+) Her-2+ tumor cells could significantly increase the PD-1 expression in CAR+ CD8+ T cells in a syngeneic mouse model, implying that PD-1 blocking antibody could potently increase CAR-T therapy [106]. Indeed, the PD-1-based inhibitory chimeric antigen receptors (iCARs) exhibited an ability to selectively suppress the function of endogenous T cell receptor (TCR) or CAR-T cells. Therefore, blockading PD-1 signaling in combination with CAR-T cell therapy may potentiate the therapeutic efficacy by overcoming the PD-L1+ tumor-mediated immunosuppressive effect. This notion was supported by a recent study in the effect of PD-L1 expression of tumor cells on CAR-T function [102]. Results from this study showed that the PD-L1 expression of tumor cells suppressed the 4-1BB*ζ* CAR-T cell function of tumor clearance in a xenograft model, and a disruption of PD-1 by CRISPR/Cas9-mediated gene editing within CAR-T cells led an augmented CAR-T cell antitumor efficacy. This study thus indicates a promise of CRISPR/Cas9-mediated PD-1 disruption of CAR-T cells for enhancing immunotherapeutic efficacy of CAR-T therapy, which also suggests that the precision genome editing of CAR-T and PD-1 is the next generation of cell therapies (Figure 2) [102].

## 6. The Challenge of Resistance to Anti-PD-1/PD-L1 Therapy

Immune checkpoint blockades have displayed a great potential in cancer therapy, which exhibited a remarkable efficacy compared to conventional treatment for advanced NSCLC [11, 107]. To date, several monoclonal antibodies (mAbs) to PD-1/PD-L1 or CTLA-4 are approved by FDA for cancer treatments. However, not all patients always benefit from these agents [14], and the data of preclinical and clinical trials demonstrated that only 20–50% patients benefited from anti-PD-1/PD-L1 therapy for various cancer types [14, 51, 52]. Equally important, the development of resistance to anti-PD-1/PD-L1 immunotherapy further leads to the failure and poor prognosis in advanced NSCLC patients receiving anti-PD-1/PD-L1 treatment. However, the mechanism underling the resistance is not fully understood. In addition, little is known about the contributions of exceeding the PD-L1-positive level, the potential antigen load or mutational load in the tumor and the genetic determinants to efficacy, and resistance to anti-PD-1/PD-L1 therapy [35].

There are several lines of studies in investigating mechanisms of therapeutic resistance to anti-PD-1/PD-L1 agents. A study by Koyama et al. found that an upregulation of T cell immunoglobulin-3 (TIM-3) in T cells could increase the adaptive resistance to therapeutic PD-1 blockade in fully immunocompetent mouse models of lung adenocarcinoma. Conversely, the sensitivity of anti-PD-1 therapeutic blockade could be restored in these mouse models after addition of anti-TIM-3 antibodies [108]. These results suggested that a strategy by downregulating TIM-3 might enhance the sensitivity of cancer cells to therapeutic PD-1 blockade. More recently, Anagnostou et al. unraveled that the evolving landscape of tumor neoantigens in NSCLC patients who were treated with immune checkpoint inhibitors (ICIs) initially respond and emerge the acquired resistance [43]. In resistant tumor clones, the authors found that 7 to 18 putative mutation-associated neoantigens were lost and the loss of neoantigens occurred either by an elimination of tumor subclones or by a deletion of truncal chromosome regions [43]. Intriguingly, these new mutations did not encode new antigens, suggesting that an expansion of the breadth of new antigenic reactivity could reduce the development of acquired resistance [43]. In addition, an acquired resistance to EGFR-TKIs or cytotoxic drugs (cisplatin or vinorelbine) could induce a downregulation of E-cadherin and alter PD-L1 expression in lung cancer cells [109]. These studies suggest a necessity to investigate the mechanism of acquired resistances to immune checkpoint blockades.

## 7. Conclusion

Conventional cancer treatments, including surgical resection, chemotherapy, and/or radiotherapy, have shown modest progress in NSCLC survival over the past two decades. The introduction of targeting agents, such as EGFR-TKI or ALK inhibitor, further offered significant improvements in NSCLC survival carrying an *EGFR* mutation or *ALK* rearrangement. To date, there are four anti-PD-1/PD-L1 agents including pembrolizumab (Keytruda), nivolumab (Opdivo), atezolizumab (Tecentriq), and durvalumab (Imfinzi) have been licensed for serving as first- or second-line NSCLC therapy. And other agents, such as MEDI0680, PDR001, REGN2810, BMS-936559, and avelumab (Bavencio) have been entered in preclinical or clinical trials for NSCLC treatment. Therefore, we expect that these agents can improve clinical efficacy and are approved for NSCLC therapy in practice. Indeed, a recent meta-analysis of five trials demonstrated that nivolumab and pembrolizumab were correlated with a significant increase of ORR as compared to atezolizumab, while nivolumab was found to associate with a lower incidence of G3-5 AEs in comparison with other anti-PD-1/PD-L1 agents for NSCLC treatment [110].

However, there are grave challenges in anti-PD-1/PD-L1 therapies for NSCLC patients. These include how to identify patients who may benefit from the therapy and minimize the development of acquired resistance to the therapy. Therefore, with an ultimate goal toward the improvement of the therapeutic efficacy and reduction of the adverse effects of anti-PD-1/PD-L1 blockades, continuing efforts are required to identify novel predictive biomarkers for patient selection by taking advantage of the rapid development of computational models and high-throughput sequencing techniques for effective and personalized immunotherapy. In addition, with our deeper understanding of mechanisms of immune escape and its role in the biological behaviors of NSCLC, a combinatory approach on the basis of anti-PD-1/PD-L1 therapy, such as a combination with chemotherapy, targeted therapy, radiotherapy, and other immunotherapies, may establish landmarks for treatment of NSCLC. For instance, in NSCLC patients with acquired resistance to anti-PD-1/PD-L1 immunotherapies and *EGFR* or *ALK* mutations, particularly for patients with high tumor burden or rapid disease progression, a combination of anti-PD-1/PD-L1 therapy and targeted therapy may be an option. To this end, it is important to comprehend mechanisms of the resistance to anti-PD-1/PD-L1 agents and identify patients who may potentially benefit from therapeutic schedule. In this respect, the discovery of novel biomarkers and/or development of precise companion diagnostic assays become critical for patient selection. Currently, however, the expression of PD-L1 is only the tip of the iceberg in the predictive index of anti-PD-1/PD-L1 therapy; it is necessary to combine the multiple indexes to make the best predictive ability, indicating an importance of personalized biomarkers in guiding anti-PD-1/PD-L1 immune checkpoint blockade therapy for NSCLC.

## Figures and Tables

**Table 1:** The PD-1/PD-L1 inhibitors licensed for clinical use or under clinical trials for 1062 NSCLC treatment.

Checkpoint	Blocking agent	IgG isotype and characteristics	Clinical stage	Manufacturer
PD-1	Pembrolizumab (MK3475, Keytruda, lambrolizumab)	Humanized IgG4 mAb	EMA, FDA approved for second-line NSCLC treatment	Merck
	Nivolumab (BMS936558, Opdivo, MDX-1106, ONO-4538)	Fully human IgG4 mAb	FDA approved for first-line and second-line NSCLC	Bristol-Myers Squibb
	MEDI0680 (AMP-514)	Humanized IgG4 mAb	Phase I	Medimmune
	PDR001	Humanized IgG4 mAb	Phase I	Novartis
	REGN2810	Humanized IgG4 mAb	Phase I	Regeneron-Sanofi

PD-L1	Atezolizumab (Tecentriq, MPDL3280A, RG7446)	High-affinity human IgG1	FDA approved for second-line NSCLC	Genentech/Roche
	Durvalumab (MEDI4736, Infinzi)	Human IgG1-*κ* mAb	FDA approved for treatment of unresectable stage III NSCLC without relapse after platinum-based chemoradiation	MedImmune/AstraZeneca
	BMS-936559 (MDX1105)	Fully high-affinity human IgG4	Phase I	Bristol-Myers Squibb
	Avelumab (Bavencio, MSB0010718C)	Fully human IgG1 mAb	FDA-approved treatment for metastatic MCC	Merck Serono

FDA: Food and Drug Administration; Ig: immunoglobulin; mAb: monoclonal antibody; NSCLC: non-small-cell lung cancer; PD-1: programmed death-1; PD-L1: programmed death-ligand 1; PD-L2: programmed death-ligand 2.

**Table 2:** PD-L1 IHC assay systems as companion and complementary diagnostic assays for NSCLC treatment using anti-PD-1/PD-L1 agents.

Assay system	Source and antibody clonality	Therapeutic antibody	Type of tissue	Detection systems required	Instrument	TPS	Company	Cancer
PD-L1 IHC 28-8 pharmDx Dako	Rabbit monoclonal	Nivolumab	FFPE	Autostainer Link 48	EnVision FLEX visualization system	Tumor cell membrane staining	Dako Autolink 48	Nonsquamous NSCLC
PD-L1 IHC 22C3 pharmDx Dako	Mouse monoclonal	Pembrolizumab	FFPE	Autostainer Link 48	EnVision FLEX visualization system	Tumor cell membrane staining	Dako Autolink 48	NSCLC
Ventana PD-L1 (SP142) assay	Rabbit monoclonal	Atezolizumab	FFPE	OptiView Amplification	Ventana BenchMark ULTRA	Tumor cell membrane and immune cell staining	Ventana Ultra	NSCLC
Ventana PD-L1 (SP263)	Rabbit monoclonal	Durvalumab	FFPE	OptiView Amplification	Ventana BenchMark ULTRA	Tumor cell membrane staining	Ventana Ultra	NSCLC

FFPE: formalin-fixed paraffin-embedded; IC: immune cells; NSCLC: non-small-cell lung cancer; PD-1: programmed death-1; PD-L1: programmed death-ligand 1; TC: tumor cells; TPS: tumor proportion score.

**Table 3:** Clinical trials of combination therapy with anti-PD-1/PD-L1 and targeted agents for NSCLC treatment.

PD-1/PD-L1 inhibitor	Targeted agent	Patients enrolled	Clinical setting(s)	Design and status	Phase	Clinical trial (NCT#)	Status	Estimated completion date
Pembrolizumab	Afatinib	38	Stage IIIA/IIIB/IV NSCLC (EGFR+) with resistance to erlotinib	Afatinib dimaleate (first) + pembrolizumab versus pembrolizumab (first) + afatinib dimaleate. Patients: recruiting participants.	I/Ib	NCT02364609	Recruiting	December 2018
Durvalumab	Gefitinib	56	NSCLC (EGFR+)	Durvalumab + gefitinib	I	NCT02088112	Active, not recruiting	June 14, 2019
Atezolizumab	Rociletinib (CO1686)	3	Advanced/metastatic NSCLC (EGFR+) regardless of T790M mutation	Rociletinib + atezolizumab	Ib/2	NCT02630186	Active, not recruiting	January 2017
Durvalumab	Osimertinib (AZD9291)	298	Advanced NSCLC (EGFR+)	AZD9291 + AZD6094 versus AZD9291 + continuous selumetinib versus AZD9291 + intermittent selumetinib versus AZD9291 + MEDI4736 versus AZD9291 + AZD6094 versus AZD9291 + selumetinib versus AZD6094 monotherapy (Japan only)	I	NCT02143466		December 28, 2018
Ipilimumab or nivolumab	Erlotinib or crizotinib	14	EGFR+ or ALK+ stage IV NSCLC	Erlotinib + erlotinib versus ipilimumab + crizotinib versus erlotinib + nivolumab versus crizotinib + nivolumab	I	NCT01998126	Active, not recruiting	December 2017
Atezolizumab	Erlotinib/alectinib	52	NSCLC (EGFR+ or ALK+)	Stage 1: alectinib + atezolizumab versus erlotinib + atezolizumab Stage 2: alectinib + atezolizumab versus erlotinib + atezolizumab	Ib	NCT02013219	Active, not recruiting	December 1, 2018
Nivolumab	Crizotinib	78	NSCLC (ALK+)	Ceritinib + nivolumab	I	NCT02393625	Recruiting	October 2017
Pembrolizumab	Crizotinib	70	Advanced NSCLC (ALK+)	Crizotinib plus pembrolizumab	Ib	NCT02511184	Recruiting	September 2018
Nivolumab	Bevacizumab	472	Advanced NSCLC with response to platinum based chemotherapy	Nivolumab alone or +bevacizumab for maintenance treatment	I	NCT01454102	Recruiting	December 2018
Pembrolizumab	Ramucirumab	155	Solid tumor including NSCLC	Ramucirumab + pembrolizumab	Ia	NCT02443324	Active, not recruiting	August 2019
Pembrolizumab	Nintedanib	18	Solid tumor including NSCLC	Nintedanib + pembrolizumab	I	NCT02856425	Recruiting	July 2021

ALK: anaplastic lymphoma kinase; EGFR: epidermal growth factor receptor; NSCLC: non-small-cell lung cancer; PD-1: programmed death-1; PD-L1: programmed death-ligand 1. Information was summarized based on data published in https://www.clinicaltrials.gov/.

## Abbreviations

ADC: Adenocarcinoma
AEs: Adverse events
AKL: Anaplastic lymphoma kinase
APC: Antigen-presenting cell
BATF: Basic leucine transcription factor, ATF-like
BCR: B cell receptor
CAR: Chimeric antigen receptor
CCRT: Concurrent chemoradiation therapy
CK2: Casein kinase 2
CRC: Colorectal cancer
CTLA-4: Cytotoxic T-lymphocyte-associated antigen 4
DAG: Diacylglycerol
DC: Dendritic cells
E2: Estrogen 2
EGFR: Epidermal growth factor receptor
EGFR-TKIs: Epidermal growth factor receptor tyrosine kinase inhibitors
ERK: Extracellular signal-regulated kinase
IC: Immune cells
iCARs: Inhibitory chimeric antigen receptors
ICIs: Immune checkpoint inhibitors
ITIM: Immunoreceptor tyrosine-based inhibitory motif
ITSM: Immunoreceptor tyrosine-based switch motif
GM-CSF: Granulocyte-macrophage colony-stimulating factor
MEK: Mitogen-activated and extracellular signal-regulated kinase
NK: Natural killer cell
MSLN: Mesothelin
NSCLC: Non-small-cell lung cancer
ORR: Overall response rate
ORRs: Objective response rates
OS: Overall survival
PD-1: Programmed death-1
PD-L1: Programmed death-ligand 1
PFS: Progression-free survival
PLC-*γ*1: Phospholipase C gamma 1
PI3K: Phosphatidylinositol 3-kinase
RCC: Renal cell carcinoma
RFS: Recurrence-free survival
PTEN: Phosphatase and tensin homolog
scFv: Single chain variable fragment
SHP-2: Src homology 2-domain-containing tyrosine phosphatase 2
SHP-1: Src homology region 2 domain-containing phosphatase-1
TC: Tumor cells
TCR: T cell receptor
TIM-3: T cell immunoglobulin mucin-3
TMB: Tumor mutation burden
TTP: Time to progression.
